# Supplementary figures and images for: High-density DArT-based SilicoDArT and SNP markers for genetic diversity and population structure studies in cassava (Manihot esculenta Crantz)
Source: PLoS One. 2021 Jul 27;16(7):e0255290. doi: 10.1371/journal.pone.0255290 (PMC8315537; doi:10.1371/journal.pone.0255290)

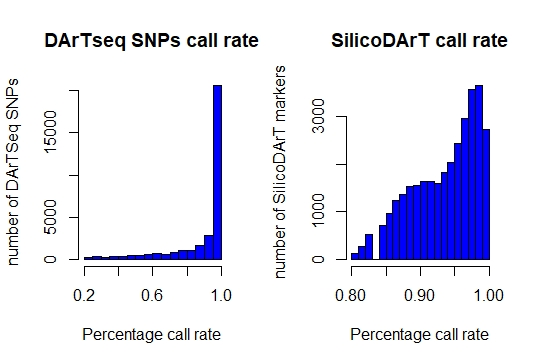

Supplement: S1 Fig — (TIF) [file pone.0255290.s001.tif]

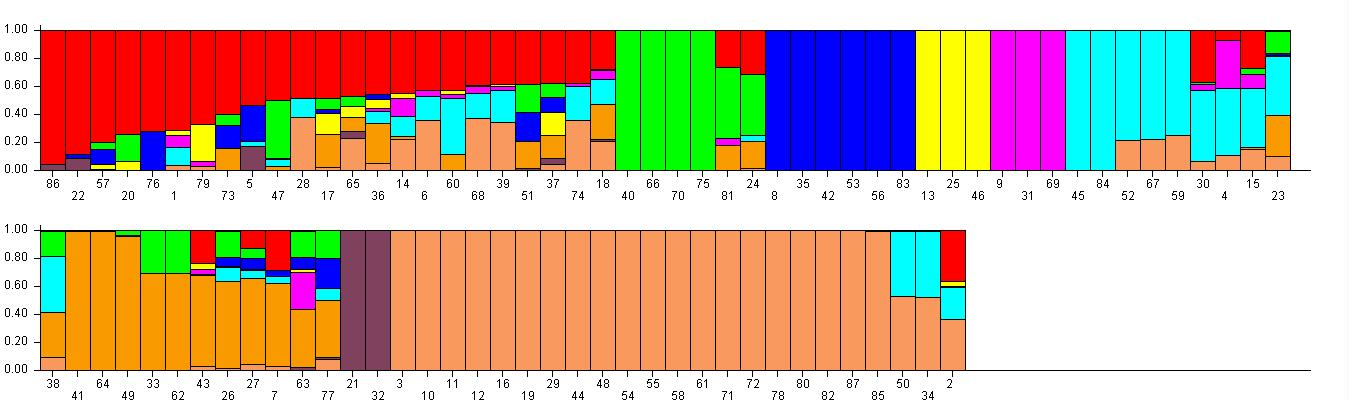

Supplement: S2 Fig — (TIF) [file pone.0255290.s002.tif]

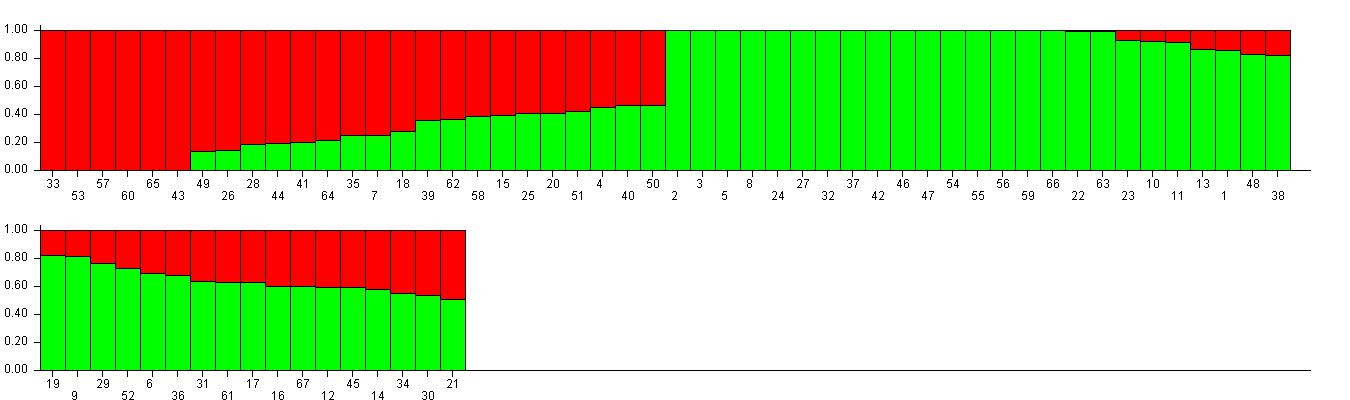

Supplement: S3 Fig — (TIF) [file pone.0255290.s003.tif]

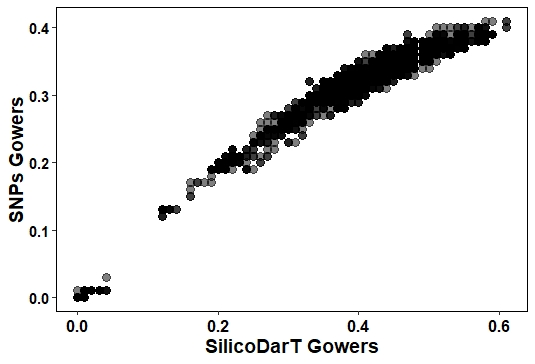

Supplement: S4 Fig — (TIF) [file pone.0255290.s004.tif]
