# Supplementary material for: High-density DArT-based SilicoDArT and SNP markers for genetic diversity and population structure studies in cassava (Manihot esculenta Crantz)
Source: PLoS One. 2021 Jul 27;16(7):e0255290. doi: 10.1371/journal.pone.0255290 (PMC8315537; doi:10.1371/journal.pone.0255290)
Supplement: Attachment — Submitted filename: Response to Reviewers.docx [file pone.0255290.s010.docx]

**Response to editor and reviewer's comments.**

| **Comments** | **Response** |
| --- | --- |
|  |  |
| **Editor’s comment:** |  |
| 1. Journal Requirements:  Please review your reference list to ensure that it is complete and correct. If you have cited papers that have been retracted, please include the rationale for doing so in the manuscript text, or remove these references and replace them with relevant current references. Any changes to the reference list should be mentioned in the rebuttal letter that accompanies your revised manuscript. If you need to cite a retracted article, indicate the article’s retracted status in the References list and also include a citation and full reference for the retraction notice | The authors have taken them into account.  7. Okogbenin, Fragene was removed based on the previous suggestion by reviewer #1 (it was outdated).  9. Swaminathan MS was inserted following the review of literature  51. Maechler M, Rousseeuw P, Struyf A, Hubert M, Hornik K. (2013) was inserted as observantly pointed out by reviewer #1.  68. Ferguson ME, Shah T, Kulakow P, Ceballos H. A global overview of cassava genetic diversity. PLoS ONE. 2019 was inserted after reviewing the document as suggested by reviewer #1. |
| While revising your submission, please upload your figure files to the Preflight Analysis and Conversion Engine (PACE) digital diagnostic tool, https://pacev2.apexcovantage.com/. PACE helps ensure that figures meet PLOS requirements. To use PACE, you must first register as a user. Registration is free. Then, login and navigate to the UPLOAD tab, where you will find detailed instructions on how to use the tool. If you encounter any issues or have any questions when using PACE, please email PLOS at figures@plos.org. Please note that Supporting Information files do not need this step. | The authors have taken them into account.  The dimensions and resolution of the figures have been improved with Picture Analysis and Conversion Engine (PACE) to meet PLOS standards. |
|  |  |
| **Reviewer #1** |  |
| General: The manuscript still requires a major English revision | The authors have taken them into account. |
| In. 33. Use expansion of PIC on first use, not on second use. | It has been addressed on line 34. |
| Ln 55. They are not tuberous roots, they are storage roots. Tubererous roots can sprout from eyes in the roots. Cassava roots do not do this, and are referred to as storage roots. | It has been addressed on line 55. |
| Ln 56 continue, not continuous | It has been addressed on line 56. |
| Ln 92. These are SNP markers visualized using DArTSeq technology. At least they should be called DArTSeq SNP markers, not just DArT markers. | The authors tried to shed a brief light on the classical DArT markers first applied in rice by Daccoud et al 2001. Referring to classical DArT markers) |
| Ln 105, what do you mean 1,000 candidate polymorphic clones. Are these distinct cassava clones. If they are, then why are they candidate? All cassava clones are polymorphic, so not necessary to state this. | Corrected on line 105. |
| 159, 160: no need to repeat ‘polymorphic information content’ once it has been sued once. Also repeated on line 204. | Corrected throughout. |
| Ln 292 How did you calculate Net nucleotide distance (add to methodology) and give a reference | It was an output from the Structure simulation analysis which has been referenced on line 179. |
